# Hollow Fiber Membranes of Blends of Polyethersulfone and Sulfonated Polymers

**DOI:** 10.3390/membranes8030054

**Published:** 2018-08-02

**Authors:** Nazia Noor, Joachim Koll, Nico Scharnagl, Clarissa Abetz, Volker Abetz

**Affiliations:** 1 Helmholtz-Zentrum Geesthacht, Institute of Polymer Research, Max-Planck-Str. 1, 21502 Geesthacht, Germany; noor_nazia@ymail.com (N.N.); joachim.koll@hzg.de (J.K.); clarissa.abetz@hzg.de (C.A.); 2 Helmholtz-Zentrum Geesthacht, Institute of Materials Research—MagIC, Max-Planck-Str.1, 21502 Geesthacht, Germany; nico.scharnagl@hzg.de; 3 Institute of Physical Chemistry, University of Hamburg, Martin-Luther-King-Platz 6, 20146 Hamburg, Germany

**Keywords:** hollow fiber membrane, polymer blend, polyethersulfone, polyphenylenesulfone, X-ray photoelectron spectroscopy (XPS)

## Abstract

Hollow fiber membranes (HFM) are fabricated from blend solutions of a polyethersulfone (PESU) with a sulfonated PESU (sPESU) or a sulfonated polyphenylenesulfone (sPPSU). The influence of different additives in the dope solution and different bore fluids on the HFM are studied. The addition of poly(sodium 4-styrene sulfonate) (PSSNa)/ethylene glycol (EG) to the dope solution results in an increased water flux of the HFM compared to its counterparts without this additive system. The morphology of the hollow fibers is examined by scanning electron microscopy (SEM). The inner surface of the hollow fibers is studied by X-ray photoelectron spectroscopy (XPS), and it is found that water permeation through the hollow fiber membranes is facilitated due to the change in morphology upon the addition of the PSSNa/EG additive system, but not by the presence of hydrophilic sulfonic acid groups on the membrane surface.

## 1. Introduction

Polyethersulfone (PESU) is an extensively used material for membrane fabrication by non-solvent induced phase separation (NIPS) [1,2]. The chemical, thermal, and oxidation resistance, as well as the good mechanical properties and ease of its processing make PESU an appropriate membrane material for different types of filtration techniques [3,4]. There are several strategies to improve the hydrophilicity and anti-fouling properties of PESU [1,5]. Among them, blending sulfonated polymers into the membrane-formulating dope solution comes with some advantages, including an ease of membrane fabrication. Associating sulfonated polymers as one of the blend components in the membrane-forming solutions has been studied to increase hydrophilicity, to lower the fouling in ultrafiltration and nanofiltration membranes, and as the support layer for thin film composite membranes for forward osmosis [1,6,7,8]. Sulfonated polymers were reported as a support layer material to facilitate water transport or improve the interfacial adhesion in composite membranes [9,10] as well. Generally, the degree of sulfonation and the concentration of the sulfonated polymer in the dope solution affects the performance of a membrane [11].

In this study, we characterized and analyzed the hollow fiber membranes (HFM), which were made of a blend of PESU and sulfonated PESU (sPESU). sPESU was prepared by following one of the post-sulfonation methods reported in the literature [12]. Using additives in the polymer dope solution is a common method to alter the membrane morphology. The morphological features of the NIPS membrane, such as number density of pores, pore size distribution, pore interconnectivity, and the hydrophilicity of the membrane can be induced by the influence of additives [13]. Poly(ethylene glycol) (PEG), polyvinylpyrrolidone (PVP), and γ-butyrolactone (GBL) are widely used additives that have been studied for the modification of the structure of NIPS membranes [1,13,14,15,16,17,18]. These additives were added to the dope solutions of PESU/sPESU, and the effect on the hollow fiber membranes was analyzed. Different bore fluids were employed as well to spin the PESU/sPESU hollow fiber membranes. The water permeance of the hollow fiber membranes was measured and discussed in light of their morphological and surface features. Another set of hollow fiber membranes was fabricated where commercially available sulfonated polyphenylenesulfones (sPPSU) were used as a blend partner with PESU in the dope solution. sPPSU was synthesized by following the direct sulfonation route, which is described by Widjojo, et al. [8]. The morphological features, surface properties, and the performance of the PESU/sPESU membranes were compared with those of the PESU/sPPSU membranes.

Addition of the polyelectrolyte poly(sodium 4-styrene sulfonate) (PSSNa) to the membrane-forming dope solution was previously reported to improve the anti-fouling property of an anion exchange membrane or to modify the permselectivity of a nanofiltration membrane [19,20]. In this work, we investigated the effect of PSSNa/ethylene glycol (EG) addition on the PESU/sulfonated polymer blend hollow fiber membranes. The water flux of the hollow fiber membranes prepared from the dope solutions with and without PSSNa/EG is discussed together with their morphology. Elemental analysis of the inner surface of the hollow fibers was performed to examine the dominating factor influencing the permeance through these hollow fiber membranes.

## 2. Materials and Methods

### 2.1. Materials and Reagents

Polyethersulfone (PESU) (Ultrason^®^ E6020P), sulfonated polyphenylenesulfone with a sulfonation degree of around 5 mol % and 8.4 mol % (referred to as sPPSU5 and sPPSU8.4, respectively) were received from BASF (Ludwigshafen, Germany). Chlorosulfonic acid (99%), poly(sodium 4-styrene sulfonate) (PSSNa) (Mw ~ 70,000, powder), and glycerol (anhydrous ≥99%) were purchased from Sigma-Aldrich (St. Louis, MO, USA). Dichloromethane (DCM), *N*-*N*-dimethylformamide (DMF), dimethyl acetamide (DMAc), 1-methyl-2-pyrrolidone (NMP), γ-butyrolactone (GBL), ethylene glycol (62.07 g/mol), and poly(ethylene glycol) (PEG200) were purchased from Merck, Germany. Polyvinylpyrrolidone (PVP K15 Mw 10,000) was procured from Fluka (Buchs, Switzerland). Water with pH values of 2 and 11 were prepared by using Titrisol buffer concentrate (Merck, Darmstadt, Germany).

### 2.2. Sulfonation of PESU

Polyethersulfone (PESU) (Ultrason^®^ E6020P) was sulfonated by following a procedure described in the literature [12]. For this reaction, dichloromethane (DCM) and 99% chlorosulfonic acid (ClSO_3_H) were chosen as a solvent and as a sulfonating agent, respectively. The degree of sulfonation (DS) was maintained at around 10% by adjusting the amount of sulfonating agent added and the reaction time. The characterization spectra and the calculation to determine the DS of this polymer are shown in the supporting information (Figure S1). The resulted polymer is termed as sPESU10 in this study.

### 2.3. Hollow Fiber Membranes

#### 2.3.1. Fabrication of the Hollow Fiber Membranes

Polymer (in case of PESU membrane) or polymers (in case of PESU and sulfonated polymer blend membranes) was/were dissolved in a solvent or in a mixture of solvent and additive to prepare the spinning dopes. Polymer solutions were stirred in sealed glass bottles overnight. After complete dissolution, the solutions were left overnight without agitation to remove air bubbles. Water or a water/glycerol mixture was used as a bore fluid. The water/glycerol mixture was prepared by stirring the components in capped bottles. The designated containers for polymer solution and bore fluid were filled with the respective solutions, and then the solutions were pumped to the designated orifices of the spinneret by using a gear or an infusion pump. In case of using the gear pump, the flow rates were determined in the weight of the solution coming out of the pump in a minute, i.e., g/min. In case of using the infusion pump, the flow rates were determined in the volume of the solution pressed through the orifice in one minute i.e., mL/min.

**Figure 1:**
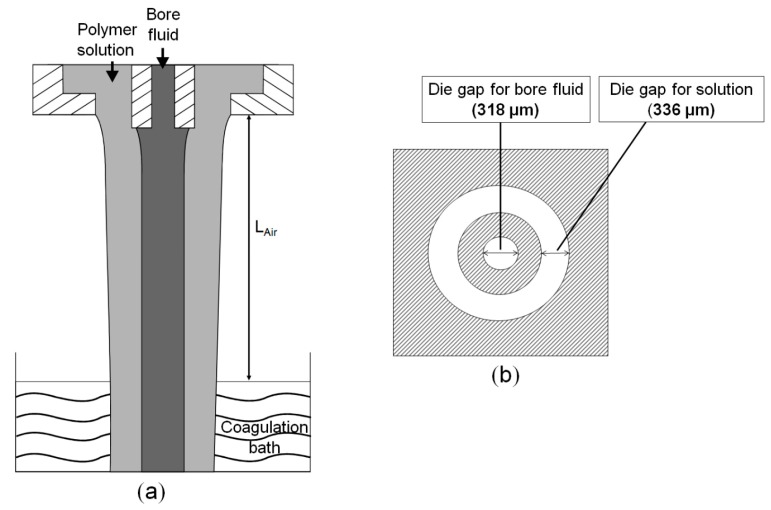
(**a**) Spinning of hollow fiber membrane by non-solvent induced phase separation (NIPS). Polymer solution and bore fluid are extruded simultaneously from a double-orifice spinneret. Fiber enters the coagulation bath following a definite air gap distance, L_Air_; (**b**) Schematic representation of the exit of the spinneret used in the experiments.

The hollow fibers were spun by the dry-jet wet spinning method where the dope solution was extruded through the spinneret together with the bore fluid [21]. The spun fibers entered into the coagulation bath (water) after experiencing a definite air gap distance (L_Air_) where the solid hollow fibers formed by non-solvent induced phase separation (NIPS). The schematic representations of the hollow fiber spinning method and the dimensions of the spinneret exit are shown in Figure 1.

#### 2.3.2. Compositions of the Solutions for Hollow Fiber Spinning

Different dope solutions were prepared to spin the hollow fiber membranes. In the first set of experiments, commonly used additives were mixed in the dope solutions. The concentrations and the compositions of the solutions involved in this set of experiments are listed in Table 1 with the membrane codes. The spinning parameters of this set of hollow fibers are listed in Table S1.

In another set of experiments, different bore fluids were used to spin the hollow fiber membranes. The concentrations and compositions of the solutions with their membrane codes are listed in Table 2. The spinning parameters of this set of hollow fibers are listed in Table S1.

The surface properties of a PESU/sPESU10 hollow fiber membrane was compared with a PESU/sPPSU5 hollow fiber membrane. The concentrations and the compositions of the solutions involved in the spinning of sPPSU5 containing hollow fiber membrane are shown with its membrane code in Table 3. The spinning parameters of this hollow fiber are listed in Table S1.

In order to study the effect of PSSNa/EG addition on the dope solutions, hollow fibers were spun with and without this additive system. The dope solutions for hollow fiber spinning were divided in three categories where three different polymer or polymer blend compositions were chosen, such as: (i) PESU containing dope solutions with (HF-PESU-P/E1) and without (HF-PESU-P/E0) the additive system PSSNa/EG; (ii) PESU/sPESU10 containing dope solutions with the additive system PSSNa/EG where two different compositions of the components were chosen (HF-sPESU10-P/E1 and HF-sPESU10-P/E2); (iii) PESU/sPPSU8.4 containing dope solutions with (HF-sPPSU8.4-E/P1) and without the additive system PSSNa/EG (HF-sPPSU8.4-E/P0).

The concentrations and compositions of this set of hollow fibers are listed in Table 4, and the membrane codes and the spinning parameters are listed in Table S2.

### 2.4. Characterization of the Polymers and Hollow Fibers

#### 2.4.1. Thermal Characterization

A NETZSCH TG 209F1 Iris (Selb, Germany) was used for thermogravimetric analysis (TGA) of the polymers and the hollow fibers. The analyses were done at a heating rate of 10 °C/min from 30 °C to 800 °C. The experiments were done in Ar atmosphere at a flow rate of 20 mL/min.

#### 2.4.2. Nuclear Magnetic Resonance Spectroscopy (NMR)

The hollow fibers, sPESU10, and PSSNa were characterized by ^1^H NMR spectroscopy using a Bruker AV-300 MHz spectrometer and a Bruker Avance III HD 500 MHz NMR spectrometer (Ettlingen, Germany), respectively. In every experiment, tetramethylsilane (TMS) was used as an internal standard, and deuterated dimethyl sulfoxide (*d*_6_-DMSO) was used as a solvent to dissolve the samples in 5 mm O.D. sample tubes.

#### 2.4.3. Scanning Electron Microscopy (SEM)

The morphology of the hollow fibers (cross-section and the inner surface of the hollow fibers) were characterized by scanning electron microscopy Leo Gemini 1550VP (Zeiss, Oberkochen, Germany) at a voltage of 3–6 kV. Cross-sections of the hollow fibers were prepared by breaking them in cryogenic condition. The hollow fibers were cut longitudinally to examine their inner surfaces. All of the samples for analysis were coated with approximately 2 nm of Pt using a coating device Bal-tec MED 020 (Bal-tec/Leica Microsystems GmbH, Wetzlar, Germany).

#### 2.4.4. X-ray Photoelectron Spectroscopy (XPS)

For analyzing the chemical composition of the inner or lumen side surface of the hollow fibers, XPS was carried out by using a Kratos AXIS Ultra DLD spectrometer (Kratos, Manchester, UK) with an Al-Kα X-ray source (monochromator) operated at 225 W and under vacuum of <2.5 × 10^−9^ Torr. After degassing in the ultra-high vacuum (UHV) pre-load chamber, the hollow fibers were positioned in the UHV analytics chamber. The analyzed area was 700 µm × 300 µm. The acceleration depth was approximately 5 nm. For the region scanned, the pass energy was 20 eV, while for survey spectra, a pass energy of 160 eV was used. All of the spectra were calibrated to 284.5 eV binding energy of the C1s signal. For all of the samples, charge neutralization was necessary. The evaluation and validation of the data were carried out with the software CASA-XPS version 2.3.18. For deconvolution of the region files, background subtraction (linear or Shirley) was performed before calculation.

#### 2.4.5. Water Flux Measurement

Several pre and post-treatment methods are reported in the literature, which have different effects on the morphology of the membranes [22,23,24]. In our case, after spinning, the hollow fibers were kept in clean water for two days to remove the residual solvents and additives. After that, the water flux was measured in dead-end mode by using an in-house built automatic testing device. For water flux measurements, one hollow fiber with an effective length of 20 cm was fitted into a module for each measurement. Deionized water was fed from the lumen side of the hollow fiber, and permeate flow was received from the shell side of the hollow fiber. During the measurement, the transmembrane pressure was kept at around 1 bar. The pressure normalized permeance (P) was calculated by the following equation by normalizing the water flux by the transmembrane pressure:

$$P=\frac{V}{∆p\times t\times A}$$

where *P* is the pressure normalized permeation flux (termed as water flux in the following discussion) in L m^−2^ h^−1^ bar^−1^ of the pure water from the hollow fiber, *V* is the volume of water in L, ∆*p* is the transmembrane pressure across the wall thickness of the hollow fiber in bar, *A* is the effective area of the inner surface of the hollow fiber acting in transporting water in m^2^, and *t* is the measurement time in hours.

## 3. Results and Discussion

### 3.1. Dope Solution with Common Additives

**Figure 2:**
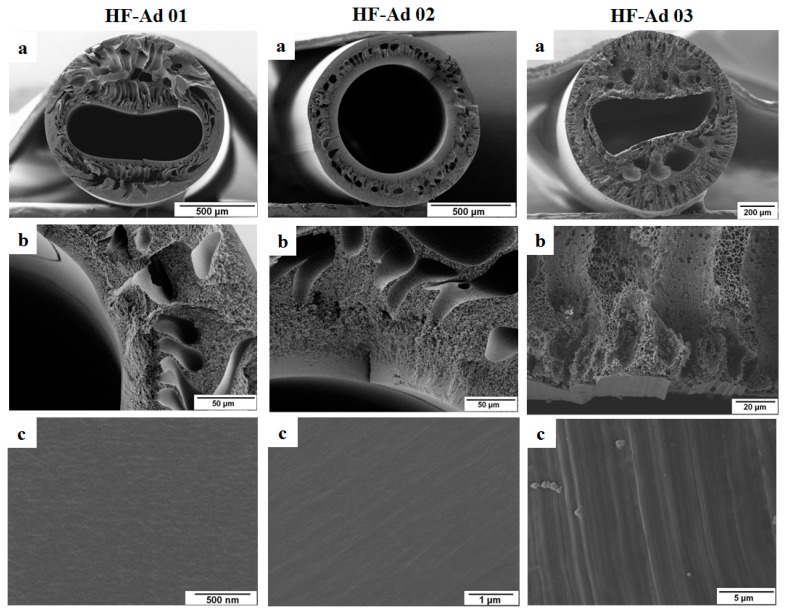
Hollow fibers HF-Ad 01, HF-Ad 02, and HF-Ad 03 (the solution compositions of these hollow fibers are listed in Table 1): (**a**) Cross-section of the hollow fiber; (**b**) Cross-sectional structure near the lumen side surface; and (**c**) Inner surface or lumen side surface of the hollow fiber.

In this section, the effect of the commonly used additives on hollow fibers (Table 1) is studied. The morphology of these hollow fibers is shown in Figure 2. The spinning dope solution was prepared by keeping a constant blend ratio for PESU and sPESU10 (60 wt % PESU and 40 wt % sPESU10). GBL, PVP, and PEG200 were used as additives in the spinning solutions of hollow fibers HF-Ad 01, HF-Ad 02, and HF-Ad 03, respectively. The morphology of the hollow fiber membranes prepared by the NIPS process was dependent on the components and composition of the dope solution and bore fluid together with the spinning parameters [25,26,27]. The SEM images in Figure 2 show that the different additives led to different morphological structures, while the concentration and composition of PESU and sPESU10 were kept constant in the solution (25% of PESU/sPESU10 (60/40) in the solution). The irregular lumen contour of the hollow fibers in our experiment resulted from the mass transfer and hydrodynamic instability as it was observed and explained in previous studies [28]. However, that discussion is out of the scope of this current study. Although the morphology of the hollow fibers HF-Ad 01, HF-Ad 02, HF-Ad 03 (fabricated from three different solution compositions) were different, the water permeance through all of them were too low to be measured. Only a few drops of water permeated through these hollow fibers after 24 h of measurement time at a transmembrane pressure of 1 bar. This circumstance may arise from the non-porous inner surface of the hollow fibers and/or the absence of pore interconnectivity.

### 3.2. Spinning with Different Bore Fluids

**Figure 3:**
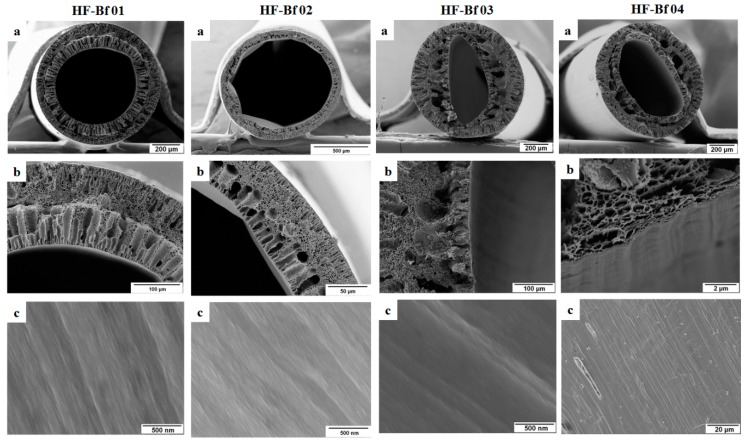
Hollow fibers HF-Bf 01, HF-Bf 02, HF-Bf 03, and HF-Bf 04 (the solution compositions of these hollow fibers are listed in Table 2): (**a**) Cross-section of the hollow fiber; (**b**) Cross-sectional structure near the lumen side surface; (**c**) Inner surface or lumen side surface of the hollow fiber.

Since the impermeability to water of the hollow fibers listed in Table 1 may arise from a higher degree of swelling of the sulfonated polymers [8], we decreased the amount of sulfonated polymer in the dope solutions. The spinning dope solution was prepared by keeping a constant blend ratio for PESU and sPESU10 (90 wt % PESU and 10 wt % sPESU10), and the hollow fibers were spun by using different bore fluids. The compositions of the spinning dopes and bore fluids are listed in Table 2. The morphological features of these hollow fibers from SEM micrographs are shown in Figure 3.

The experiments with the different bore fluids resulted in hollow fibers with a dense-looking inner surface with a thick skin layer on the lumen side. When water or water/glycerol mixture were used as a bore fluid, no open porous inner surface was created (HF-Bf 01 and HF-BF 02). Moreover, the skin layer at the lumen side turned out to be much thicker (shown in Figure S2) when the weak non-solvent glycerol was added to the bore fluid to spin the dope solution with an increased concentration of sPESU10 (compositions are listed in Table S3). This phenomenon is more likely to happen if the solvent outflow from the dope solution is faster than the inflow of non-solvent (water/glycerol) into the nascent fiber [13,29]. A closed cell-like structure was dominating in the cross-sectional area of these hollow fibers. When the weak non-solvent glycerol was replaced and the hollow fibers were spun with the bore fluids of acidic and basic water, the same scenario was reflected with a dense inner surface and a closed cell-like structure throughout the thickness of the hollow fibers. A sufficiently high polymer concentration of the spinning solution with a higher outflow of the solvent from the dope solution may follow the route to vitrify the polymers at the lumen side first, and may cause the formation of a thick layer near the inner surface. This thick inner side surface influences the solvent–non-solvent exchange during the spinning process, and the effect translates locally into a higher polymer content, which may bring the closed cell structure with a dense layer. So, the final membrane morphology is influenced by the degree of solvent outflow and non-solvent inflow during the coagulation process [28,30]. This part lets us conclude that the attempt of fabricating permeable hollow fibers was not successful, even with a lower concentration of sPESU10 in the dope solutions along with the use of different bore fluids in the spinning process.

### 3.3. Analysis of the Inner Surface of the Hollow Fibers by XPS

In this section, the inner surface of the hollow fibers is analyzed by XPS. For this study, two different hollow fibers were chosen. In one case, the inner surface of HF-Bf 01 was analyzed where sPESU10 was used as a blend component in the dope solution. In the other case, the inner surface of HF-C 01 was analyzed where a commercially available sulfonated polymer sPPSU5 was used as a blend component in the dope solution. Commercially available sPPSU polymers from BASF were synthesized from sulfonated monomers [8], unlike sPESU10, which was prepared by the post-sulfonation of PESU.

**Figure 4:**
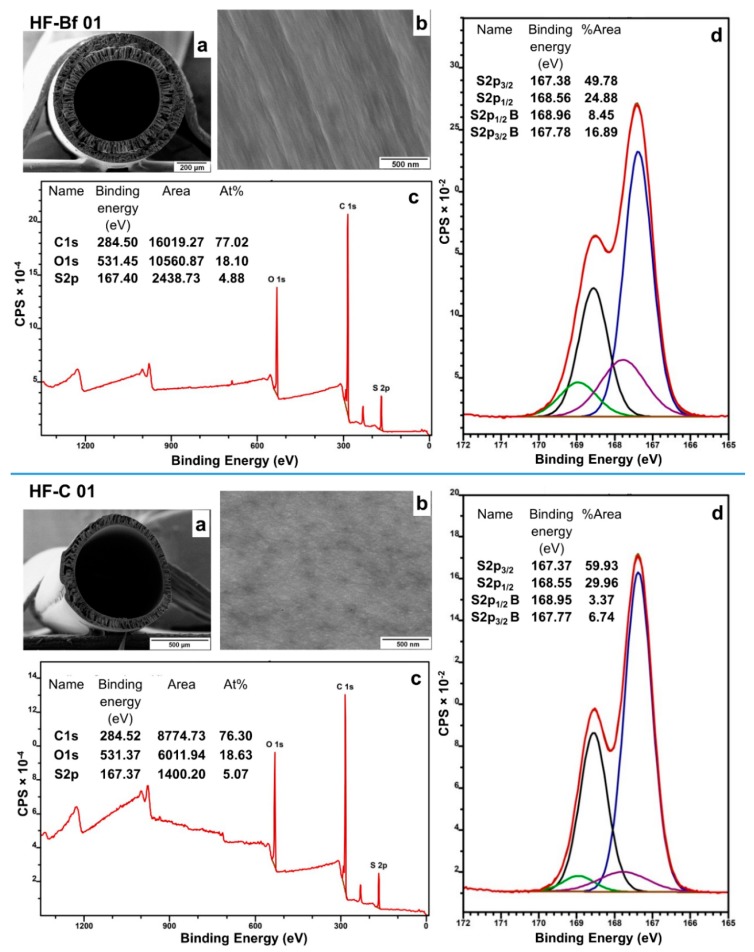
Elemental analysis of the inner surfaces of the hollow fibers HF-Bf 01 and HF-C 01. (**a**) Cross-section of the hollow fiber; (**b**) Inner surface of the hollow fiber; (**c**) X-ray photoelectron spectroscopy (XPS) survey spectrum of the components of the inner surface of the hollow fiber; (**d**) Deconvoluted S2p region from the XPS spectrum.

Core level regions from the inner surfaces were recorded at different binding energies. In our study, we validated the S2p region very carefully to avoid the wrong interpretation of S2p_3/2_ as S2p. For S2p, peak overlapping spin-orbit doublets were detected, and closely spaced spin-orbit components of S2p peak were detected at a 1.18 eV binding energy difference with an intensity ratio of around 0.5. The S2p region was deconvoluted, and the regions for two different sulfur species for sulfonyl groups (O=S=O) and sulfonic acid groups (–SO_3_H) were identified. The spectra from XPS are shown in Figure 4, and the S2p regions are given in Table 5 (peaks from the sulfur of sulfonic acid groups are termed with B). The near-surface coverage of sulfonated polymer was calculated by [S_(–SO__3H)_/S_(O=S=O)_] × 100%.

For example, S_(–SO3H)_ is the peak area at S2p_1/2_ B or at S2p_3/2_ B, and S_(O=S=O)_ is the peak area at S2p_1/2_ or at S2p_3/2_. The amount of S from (–SO_3_H) groups to S from (O=S=O) groups is calculated to be around 34% (16.89/49.78 × 100% = 33.9%, or 8.45/24.88 × 100% = 33.9%). This method is followed in every case to calculate the near-surface coverage of sulfonated polymer on the inner surface of the hollow fibers.

The comparison between the inner surface analyses of the hollow fibers HF-Bf 01 and HF-C 01 led to the conclusion that the inner surface of the hollow fiber that contained sPESU10 (HF-Bf 01) was much enriched in sulfur from the sulfonic acid groups. Surface segregation was caused by the repulsive forces between the hydrophilic chains and the hydrophobic membrane matrix as soon as the bore fluid (coagulant) came into contact with the inner surface of the hollow fiber. When the spinning solution encountered the bore fluid, which was water, hydrophilic chains of sPESU10 tended to face toward the bore fluid side i.e., accumulated at the surface of the lumen side. This phenomenon of the preferential orientation of sulfonic acid groups toward water was explained in the previous works as well, where sulfonated polymers were used as a blend partner in membrane-casting solutions [9,11]. From the analysis of HF-C 01, it was found that sulfonated chains were less abundant on the inner surface. The previous studies showed that the enrichment of sulfonic acid groups on the surface enhanced the permeation of water through the membrane, although the surface appeared non-porous. The enhanced permeance was often attributed to the increased bulk porosity and hydrophilicity [3]. Nevertheless, in our case, the hollow fiber that was spun with PESU/sPPSU5 (HF-C 01) showed some water permeance (22 L m^−2^ h^−1^ bar^−1^) in spite of having less enrichment of sulfonic acid groups on the inner surface. However, the hollow fiber spun with PESU/sPESU10 (HF-Bf 01) was impermeable. The water permeance of hollow fibers depends on the overall pore structure (pore size, porosity) and pore interconnectivity [6]. In the case of HF-Bf 01, the missing interconnectivity of the pores throughout the cross-section might be a reason for impermeability.

The permeability of HF-C 01 might be facilitated by other reasons as well. sPPSU5 is less hydrophilic compared with sPESU10 because of its lower degree of sulfonation. A higher degree of swelling in the sPESU10 containing membranes could be a big contributor to the resistance to water flow. In the previous studies, it was shown that the membranes prepared with a polymer of higher degree of sulfonation led to a decrease in water flux values due to the increased swelling effect [5,8].

### 3.4. Characterization and Performance of Hollow Fiber Membranes Prepared with PSSNa/EG in the Dope Solution

The results from the previous sections had motivated the search of an additive system that can alter the morphology of the PESU/sPESU10 hollow fibers to a permeable one. In this study, we dissolved PSSNa in ethylene glycol (EG), and incorporated this into the spinning solution to examine the effect on the final hollow fiber membranes’ performance.

**Figure 5:**
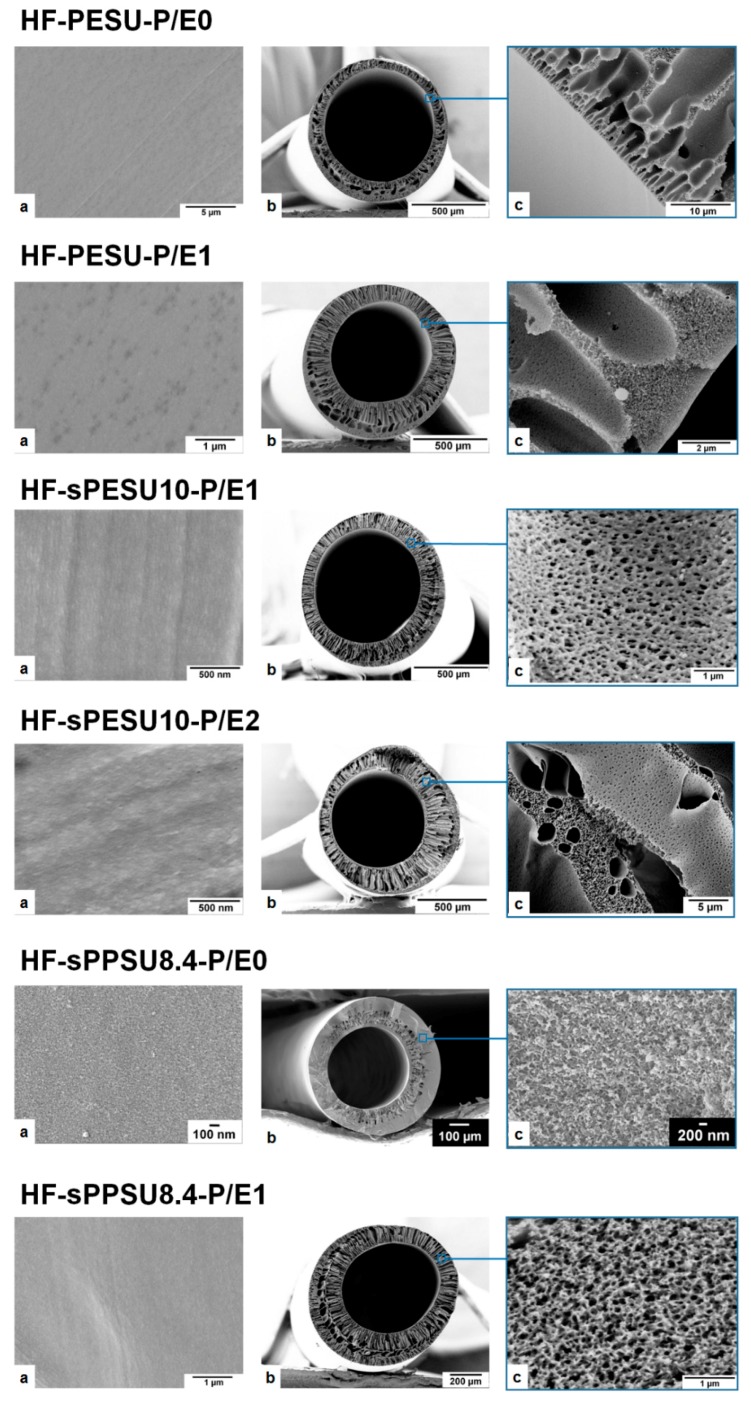
Hollow fibers spun with the dopes listed in Table 4: (**a**) Inner surface or lumen side surface of the hollow fiber; (**b**) Cross-section of the hollow fiber; (**c**) Cross-sectional structure near the lumen side surface or in the middle section of the hollow fiber.

**Figure 6:**
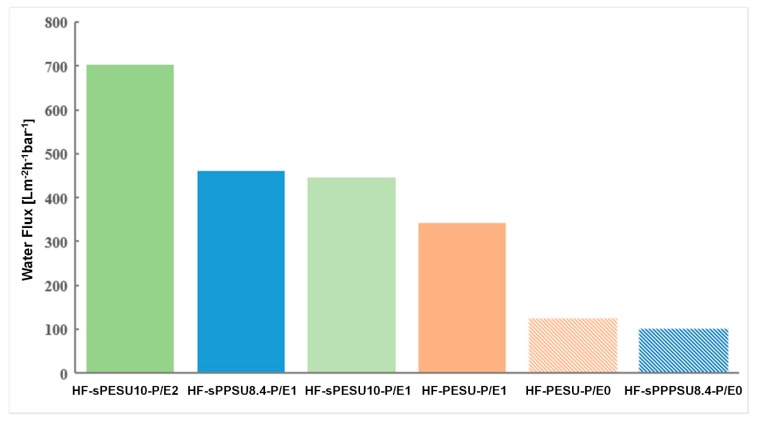
Water flux of the hollow fiber membranes spun from the dope solutions listed in Table 4 after half an hour.

The solution compositions of the hollow fibers are listed in Table 4. Morphological features from SEM are shown in Figure 5, and the results from the water flux measurements are shown in Figure 6.

In the previous sections, we have shown that the hollow fibers that were spun with PESU/sPESU10 containing dope solutions were impermeable to water. From Figure 6, it can be seen that the PESU/sPESU10 and PSSNa/EG containing dope solutions resulted in hollow fibers (HF-sPESU10-P/E1 and HF-sPESU10-P/E2) that showed high values of water flux. Moreover, from Figure 6, it is observed that the addition of PSSNa/EG in the PESU containing dope solution increased the water flux value of the hollow fiber membranes (comparison between HF-PESU-P/E0 and HF-PESU-P/E1) as well. However, in the case of PSSNa/EG addition to the PESU dope solution, the increase in water flux is not as much as in the case of PSSNa/EG addition to the PESU/sulfonated polymer blend dope solutions. In the case of PESU membranes (HF-PESU-P/E1 and HF-PESU-P/E0), the pure water flux increases to 340 L m^−2^ h^−1^ bar^−1^ from 125 L m^−2^ h^−1^ bar^−1^ upon the addition of PSSNa/EG to the PESU solution. The thickness and the morphological features of the hollow fibers play a vital role in determining the permeation or water flux of the membranes, which is also dependent on the solution system and the spinning parameters. The most notable part of the polyelectrolyte addition is that it led to the fabrication of permeable PESU/sPESU10 hollow fibers. PESU/sPESU10 (60/40) and PESU/sPESU10 (90/10) containing membranes (HF-sPESU10-P/E1 and HF-sPESU10-P/E2) showed water flux values of around 450 L m^−2^ h^−1^ bar^−1^ and 700 L m^−2^ h^−1^ bar^−1^, respectively. The effect of PSSNa/EG was also examined for a dope solution where a commercially available sulfonated polymer sPPSU8.4 was used. From Figure 6, it is noticeable that the addition of PSSNa/EG in the dope solution of HF-sPPSU8.4-P/E1 showed about a fourfold increase in the value of water flux compared with its counterpart where this additive was not used, i.e., HF-sPPSU8.4-P/E0.

It has to be noted that the total polymer concentrations were reduced in the case of PSSNa/EG additive incorporation, and this rendered into the decreased aggregation of polymer molecules through chain entanglement, which helped to increase the pore size and porosity as well [11,31]. From Figure 5, it is observed that PSSNa/EG containing dope solutions resulted in hollow fibers (HF-PESU-P/E1, HF-sPESU10-P/E1, HF-sPESU10-P/E2, and HF-sPPSU8.4-P/E1) that showed finger-like structures extended from the lumen side to the outer edge, and an interconnectivity of the porous structure throughout the cross-section.

**Figure 7:**
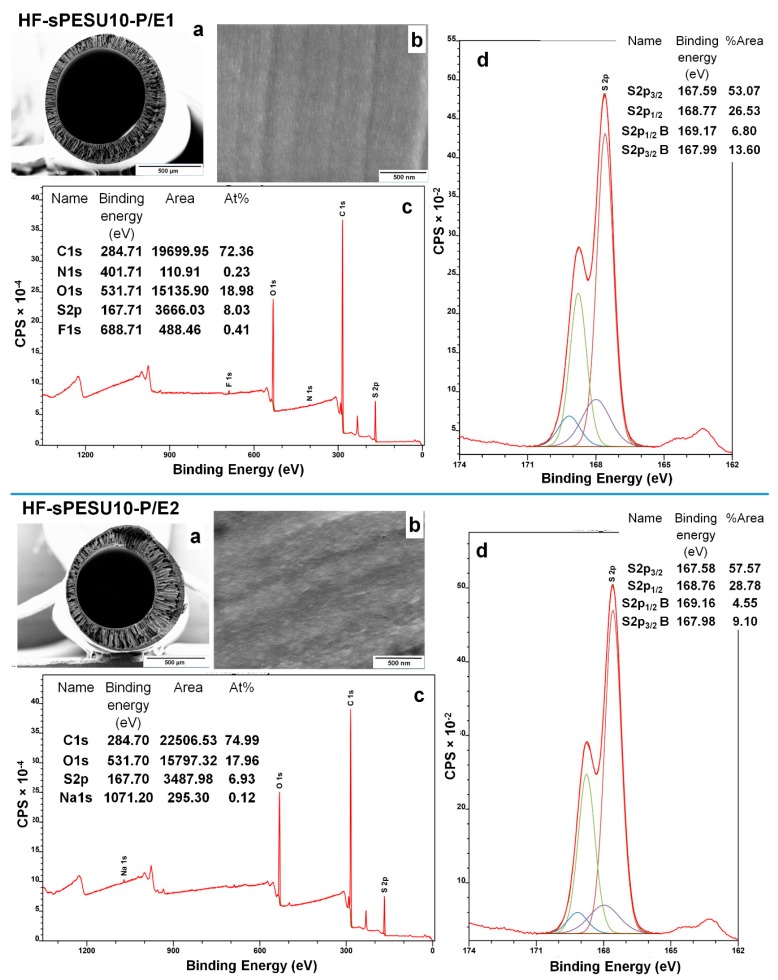
Elemental analysis of the inner surfaces of the hollow fibers HF-sPESU10-P/E1 and HF-sPESU10-P/E2: (**a**) Cross-section of the hollow fiber; (**b**) Inner surface of the hollow fiber; (**c**) XPS spectrum from the inner surface of the hollow fiber; (**d**) Deconvoluted S2p region from the XPS spectrum.

The inner surfaces of the hollow fibers HF-sPESU10-P/E1 and HF-sPESU10-P/E2 were examined by X-ray photoelectron spectroscopy (XPS). Figure 7 and Table 6 show that the near-surface coverage by the sulfonic acid groups of the hollow fiber HF-sPESU10-P/E1 is about 25.6%, and that of the hollow fiber HF-sPESU10-P/E2 is about 16%. That means that the near-surface coverage by the sulfonic acid groups was less at the inner surface of the hollow fiber that showed a higher water flux (HF-sPESU10-P/E2). So, as seen before in Section 3.3, the higher concentration of the more hydrophilic groups on the surface is less influential on water permeation in comparison to the overall pore structure and pore interconnectivity throughout the thickness of the hollow fiber. It has to be mentioned that the fluorine and nitrogen peaks from HF-sPESU10-P/E1 might have originated from the grease used in the piston to press the dope solution, and the sodium peak in HF-sPESU10-P/E2 might have come from any impurities or the additive system that was not properly washed off from the surface of this sample.

The phase inversion process of membrane formation is influenced by both thermodynamic and kinetic factors. The composition of the PSSNa/EG-associated solution and the concentration of its components were increased until the solution started to be opaque. The addition of PSSNa/EG altered the dope composition as well as the thermodynamic stability, and thus influenced the precipitation kinetics [15,32]. Previous studies showed that if PSSNa is bound to the membrane surface, then it might increase the water permeance by inducing more hydrophilicity to the membrane surface [20]. However, from our study, we assume that the PSSNa/EG additive system helped in the reorganization of the matrix-forming polymer chains during the phase inversion process. Moreover, since the polyelectrolyte PSSNa is water soluble, as is EG, due to the intrusion of water molecules from the lumen side of the hollow fiber during the spinning and from the shell side during the precipitation in the coagulation bath, PSSNa was washed away along with the low molecular weight component EG. This statement is verified by TGA and NMR measurements (shown in Figures S3 and S4).

## 4. Conclusions

In this work, PESU/sulfonated polymer blend hollow fiber membranes were evaluated in terms of their water permeance. PESU/sPESU blend solutions were spun with different additives or by using different bore fluids. PESU/sPESU containing hollow fiber membranes were found to be impermeable to water, although the higher amount of sulfonic acid groups was detected on their surface by XPS. The addition of ethylene glycol and poly(sodium 4-styrene sulfonate) to the spinning dopes of PESU/sPESU blends resulted in permeable hollow fiber membranes. By using this additive system, an increase in the water permeation of PESU/sPPSU blend hollow fibers was evident as well. Our investigation showed that the permeance of PESU/sulfonated polymer containing hollow fibers was mainly dependent on the morphological features and pore interconnectivity, rather than on the presence of hydrophilic groups on the surface.

## Figures and Tables

**Table 1:** Solution compositions of the hollow fibers.

Membrane Code	Polymer Concentration and Composition ^a^	Solvent and Additive Concentration	Bore Fluid Composition ^b^
HF-Ad 01	25% PESU/sPESU10 (60/40)	54.75% NMP 20.25% GBL	Water/NMP (80/20)
HF-Ad 02	25% PESU/sPESU10 (60/40)	45% NMP 22.5% GBL 7.5% PVP	Water/NMP (80/20)
HF-Ad 03	25% PESU/sPESU10 (60/40)	67.5% NMP 7.5% PEG200	Water/NMP (80/20)

^**a**^ Polymer concentration and composition is expressed as x% A/B (y/z), where x is the total polymer concentration in weight% in the solution, and y and z are the weight percentage of polymer A and B, respectively, in the polymer blend; ^**b**^ bore fluid composition is expressed as C/D (v/w), where v and w are the concentration of C and D, respectively, in weight% in the total bore fluid content. GBL: γ-butyrolactone; HF: hollow fiber; NMP: 1-methyl-2-pyrrolidone; PEG2000: poly(ethylene glycol); PESU: polyethersulfone; sPESU: sulfonated PESU.

**Table 2:** Solution compositions of the hollow fibers.

Membrane Code	Polymer Concentration and Composition ^a^	Solvent and Additive Concentration	Bore Fluid Composition ^b^
HF-Bf 01	25% PESU/sPESU10 (90/10)	75% NMP	Water (100)
HF-Bf 02	25% PESU/sPESU10 (90/10)	75% NMP	Water/glycerol (50/50)
HF-Bf 03	25% PESU/sPESU10 (90/10)	75% NMP	Water of pH 2 (100)
HF-Bf 04	25% PESU/sPESU10 (90/10)	75% NMP	Water of pH 11 (100)

^**a**^ Polymer concentration and composition is expressed as x% A/B (y/z), where x is the total polymer concentration in weight% in the solution, and y and z are the weight percentage of polymer A and B, respectively, in the polymer blend; ^**b**^ bore fluid composition is expressed as C/D (v/w), where v and w are the concentration of C and D, respectively, in weight% in the total bore fluid content.

**Table 3:** Solution compositions of the hollow fiber spinning.

Membrane Code	Polymer Concentration and Composition ^a^	Solvent and Additive Concentration	Bore Fluid Composition ^b^
HF-C 01	20% PESU/sPPSU5 (90/10)	80% DMAc	Water (100)

^**a**^ Polymer concentration and composition is expressed as x% A/B (y/z), where x is the total polymer concentration in weight% in the solution, and y and z are the weight percentage of polymer A and B, respectively, in the polymer blend; ^**b**^ bore fluid composition is expressed as C (w), where w is the concentration of C in weight% in the total bore fluid content. DMAc: dimethyl acetamide; sPPSU5: sulfonated polyphenylenesulfone with a sulfonation degree of around 5 mol %.

**Table 4:** Solution compositions of the hollow fiber dope solutions.

Membrane Code ^c^	Polymer Concentration and Composition ^d^	Solvent and Other Additives Concentration
HF-PESU-P/E0	18% PESU	82% NMP
HF-PESU-P/E1	18% PESU	67.9% NMP 13.4% EG 0.70% PSSNa
HF-sPESU10-P/E1	18% PESU/sPESU10 (60/40)	67.9% NMP 13.4% EG 0.70% PSSNa
HF-sPESU10-P/E2	15% PESU/sPESU10 (90/10)	59% NMP 24.7% EG 1.3% PSSNa
HF-sPPSU8.4-P/E0	25% PESU/sPPSU8.4 (60/40)	75% NMP
HF-sPPSU8.4-P/E1	18% PESU/sPPSU8.4 (60/40)	67.9% NMP 13.4% EG 0.70% PSSNa

Note: ^**c**^ Membrane codes are indicated in a way where HF-PESU indicates that the dope solution contains PESU and HF-sPESU10 or HF-sPPSU8.4 indicate that the dope solution contains PESU and a sulfonated polymer; P/E0 refers to the absence of PSSNa/EG in the solution whereas P/E1 or P/E2 refer to the presence of PSSNa/EG in the solution. ^**d**^ Polymer concentration and compositions of the polymer blends are expressed as x% A/B (y/z), where x is the total polymer concentration in weight% in the solution, and y and z are the weight percentage of polymer A and B, respectively, in the polymer blend. EG: ethylene glycol; HF-PESU-P/E0: PESU containing dope solution without the additive system PSSNa/EG; HF-PESU-P/E1: PESU containing dope solution with the additive system PSSNa/EG; HF-sPESU10-P/E1 and HF-sPESU10-P/E2: PESU/sPESU10 containing dope solutions with the additive system PSSNa/EG where two different compositions of the components were chosen; HF-sPPSU8.4-P/E0: PESU/sPPSU8.4 containing dope solution without the additive system PSSNa/EG; HF-sPPSU8.4-P/E1: PESU/sPPSU8.4 containing dope solution with the additive system PSSNa/EG; sPESU10: sulfonated polyethersulfone with a sulfonation degree of around 10 mol %; sPPSU8.4: sulfonated polyphenylenesulfone with a sulfonation degree of around 8.4 mol %.

**Table 5:** Sulfur regions from the inner surface of the hollow fibers HF-Bf 01 and HF-C 01.

Inner Surface of HF-Bf 01						Inner Surface of HF-C 01					
S 2p Region from (O=S=O) Groups			S 2p Region from (–SO_3_H) Groups			S 2p Region from (O=S=O) Groups			S 2p Region from (–SO_3_H) Groups		
Peak	Binding Energy (eV)	Area %	Peak	Binding Energy (eV)	Area %	Peak	Binding Energy (eV)	Area %	Peak	Binding Energy (eV)	Area %
S2p_3/2_	167.38	49.78	S2p_3/2_ B	167.78	16.89	S2p_3/2_	167.37	59.93	S2p_3/2_ B	167.77	6.74
S2p_1/2_	168.56	24.88	S2p_1/2_ B	168.96	8.45	S2p_1/2_	168.55	29.96	S2p_1/2_ B	168.95	3.37
Near-surface coverage of sPESU10 (% of Atoms)			**~34%**			Near-surface coverage of sPPSU5 (% of Atoms)			**~11%**		

**Table 6:** Sulfur regions from the inner surfaces of the hollow fibers HF-sPESU10-P/E1 and HF-sPESU10-P/E2.

Inner Surface of HF-sPESU10-P/E1						Inner Surface of HF-sPESU10-P/E2					
S 2p Region from (O=S=O) Groups			S 2p Region from (–SO_3_H) Groups			S 2p Region from (O=S=O) Groups			S 2p Region from (–SO_3_H) Groups		
Peak	Binding Energy (eV)	Area %	Peak	Binding Energy (eV)	Area %	Peak	Binding Energy (eV)	Area %	Peak	Binding Energy (eV)	Area %
S2p_3/2_	167.59	53.07	S2p_3/2_ B	167.99	13.60	S2p_3/2_	167.58	57.57	S2p_3/2_ B	167.98	9.10
S2p_1/2_	168.77	26.53	S2p_1/2_ B	169.17	6.80	S2p_1/2_	168.76	28.78	S2p_1/2_ B	169.16	4.55
Near-surface coverage of sPESU10 (% of atoms)			**~25.6%**			Near-surface coverage of sPESU10 (% of atoms)			**~16%**		

## Supplementary Materials

The following are available online at http://www.mdpi.com/2077-0375/8/3/54/s1. Figure S1: ^1^H NMR spectra of PESU and sPESU10, Figure S2: Hollow fiber membrane spun with the solution compositions and parameters referred in Table S3, Figure S3: TGA of polymers, PSSNa and hollow fibers, Figure S4: ^1^H-NMR spectra of PSSNa and hollow fibers, Table S1: Spinning parameters of the hollow fibers shown in Figure 2, Figure 3 and Figure 4, Table S2: Spinning parameters of the hollow fiber shown in Figure 5, Table S3: Solution compositions and the spinning parameters of the hollow fiber shown in Figure S2.
