# Supplementary material for: Hollow Fiber Membranes of Blends of Polyethersulfone and Sulfonated Polymers
Source: Membranes (Basel). 2018 Aug 2;8(3):54. doi: 10.3390/membranes8030054 (PMC6161186; doi:10.3390/membranes8030054)
Supplement: Supplementary file 1 [file membranes-08-00054-s001.pdf]

membranes

Supplementary

Hollow Fiber Membranes of Blends of Polyethersulfone and Sulfonated Polymers

Nazia Noor, Joachim Koll, Nico Scharnagl, Clarissa Abetz, and Volker Abetz

Sulfonation of PESU

Degree of sulfonation (DS) was measured by using the following equation:

$$\frac{I_b}{4I_{a1}} = \frac{1-DS}{DS} \quad (1)$$

where, I_b and I_{a1} are the integral area of peak b and a1 respectively. The calculated value of DS is 10.71%.

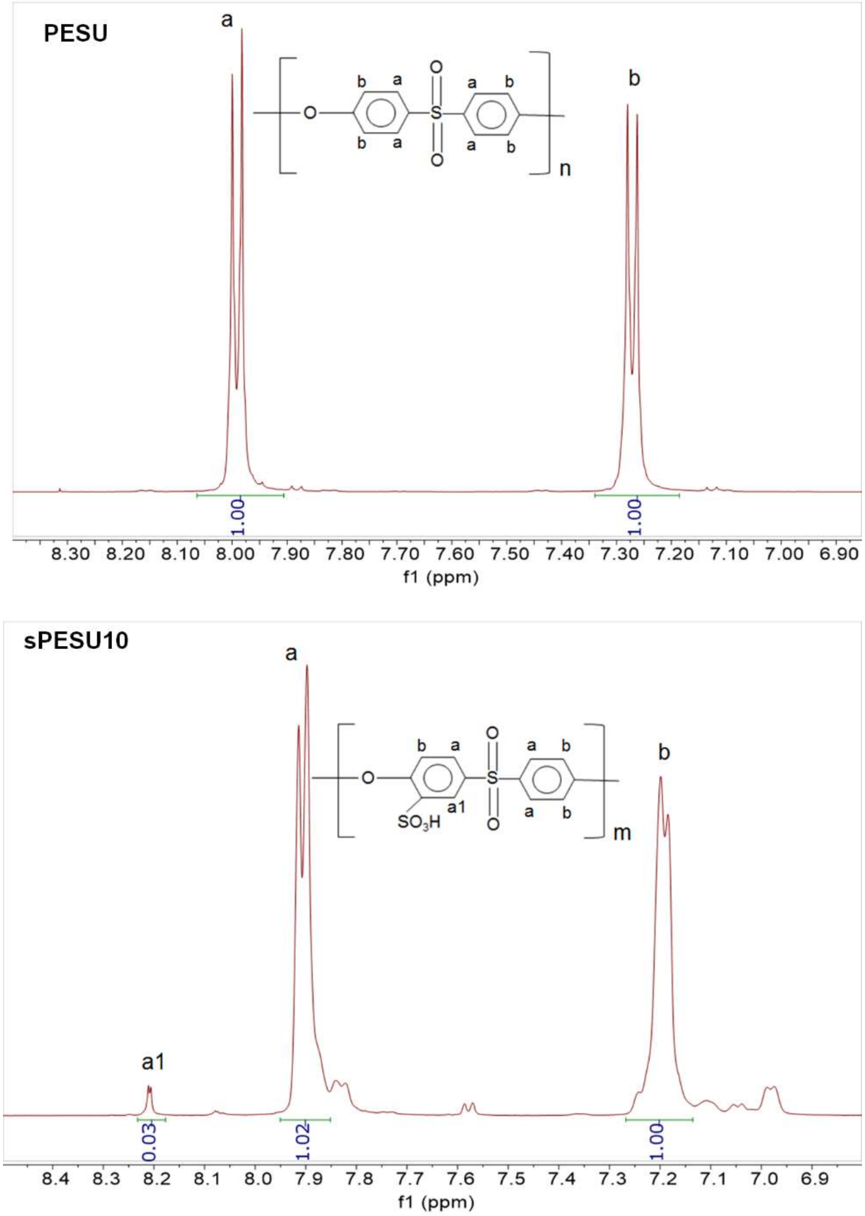

Figure S1. ^{1}H NMR spectra of PESU and sPESU10.

Table S1. Spinning parameters of the hollow fibers shown in Figure 2, 3, and 4.

Membrane ID	Flow Rate of Bore Fluid	Flow Rate of Polymer Solution	Air Gap, L_{Air}
HF-Ad 01	1.5 g/min	2.5 mL/min	10 cm
HF-Ad 02	1.5 g/min	2 mL/min	10 cm
HF-Ad 03	1.5 mL/min	2 mL/min	10 cm
HF-Bf 01	1 mL/min	0.5 mL/min	10 cm
HF-Bf 02	1 mL/min	0.5 mL/min	10 cm
HF-Bf 03	1 mL/min	1 mL/min	10 cm
HF-Bf 04	1 mL/min	1 mL/min	10 cm
HF-C 01	2 mL/min	1 mL/min	10 cm

Table S2. Spinning parameters of the hollow fibers shown in Figure 5.

Membrane Code	Flow Rate of Bore Fluid [mL/min]	Flow Rate of Polymer Solution [mL/min]	Air-gap Distance, L_{Air} [cm]	Bore Fluid and Coagulation Bath Solution
HF-PESU-P/E0<br>HF-PESU-P/E1<br>HF-sPESU10-P/E1<br>HF-sPESU10-P/E2<br>HF-sPPSU8.4-P/E0<br>HF-sPPSU8.4-P/E1	2	1	10	Water

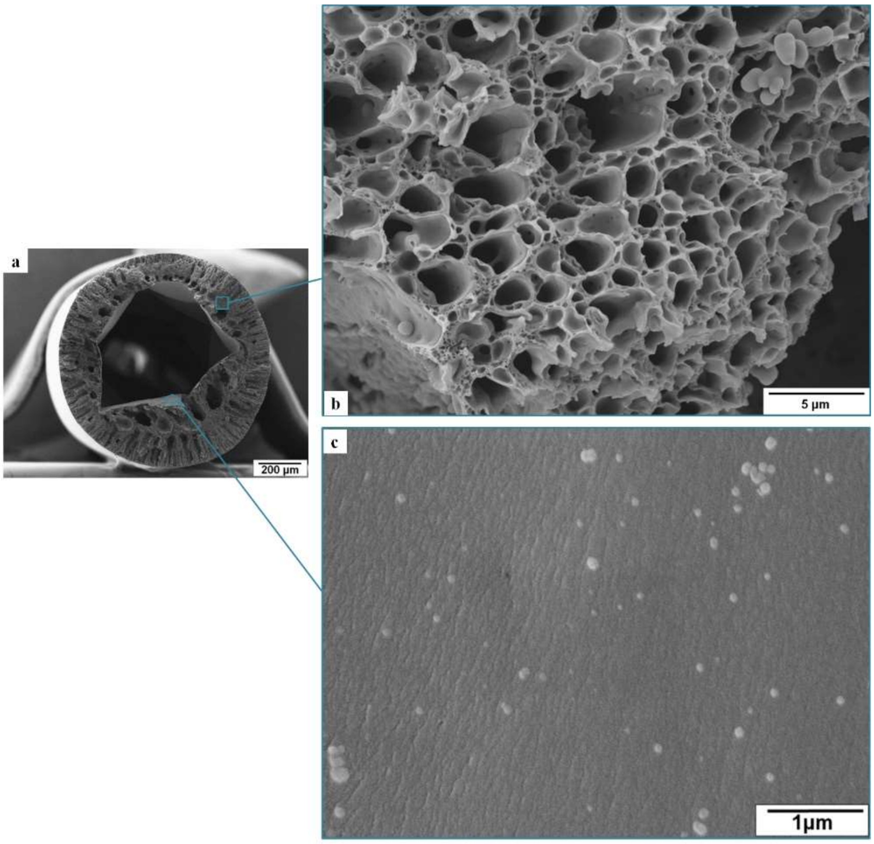

Figure S2. Hollow fiber membrane spun with the solution compositions and parameters referred in Table S3: a) Cross section of the hollow fiber, b) Magnified image at the middle area of the cross section of the hollow fiber, c) inner surface of the hollow fiber.

Table S3. Solution compositions and the spinning parameters of the hollow fiber shown in Figure S2.

Parameters	Polymer	Polymer Conc. in Total Solution	Solvent	Bore Fluid	Composition of the Bore Fluid
	PES/sPESU10 (60/40)	25%	NMP	Water/glycerol	50/50
**Flow rate**	1.5 mL/min			1 mL/min	
**Air gap, L_{Air}**			10 cm		

TGA and NMR Analysis

From Figure S3a, for the samples PES, sPESU10, HF-sPESU10-P/E1, and HF-sPESU10-P/E2 it is seen that the first step of the weight loss corresponds to the loss of water. Since the water molecules bound to the sulfonic acid groups leave at higher temperature this step extends beyond 100 ^{o}C for the samples which carry sulfonated polymers. The weight loss step at around 300 ^{o}C corresponds to the loss of sulfonic acid groups. This step is very pronounced and starts at a lower temperature for sPESU10. The weight loss observed in this step does not account for a significant mass loss for the hollow fiber HF-sPESU10-P/E1 (PES/sPESU10 (60/40) blend) and is not even noticeable for the hollow fiber HF-sPESU10-P/E2 ((PES/sPESU10 (90/10) blend). The mass loss step at around 400 ^{o}C associates with the fragmentation of the polymer main chain. This step starts at slightly lower temperature for sPESU10 and HF-sPESU10-P/E1 than that for pure PESU polymer. Samples with sulfonated polymers show lower decomposition temperatures and this is may be observed since the presence of sulfonic acid groups in the PESU structure induces enhanced asymmetry. Therefore, the less regularity in the structure brings out less stability. This phenomenon was showed in previous works as well [1-4].

In case of PSSNa, after the exclusion of water from the sample two major weight loss steps are seen at around 330 ^{o}C and 470 ^{o}C. Comparison among the mass loss spectra shows that the hollow fibers HF-sPESU10-P/E1 and HF-sPESU10-P/E2 do not retain any noticeable amount of the additive (PSSNa/EG). The comparative study on the TGA measurements of HF-sPPSU8.4-P/E0 and HF-sPPSU8.4-P/E1 is shown in Figure S3b. Here it is seen that the hollow fiber spun with PSSNa/EG containing dope solution does not show any difference in degradation behavior compared to the hollow fiber spun with a dope solution without PSSNa/EG. These results indicate that the additive system (PSSNa/EG) acts as a porogen for our system. The NMR study of the hollow fibers further affirms this observation (Figure S4). PSSNa shows three characteristic peaks at 1.7 ppm, 6.4 ppm, and 7.4 ppm. However, from Figure S4a and b it is seen that the hollow fibers spun with PSSNa/EG carrying dope solution do not show any characteristic peak of PSSNa.

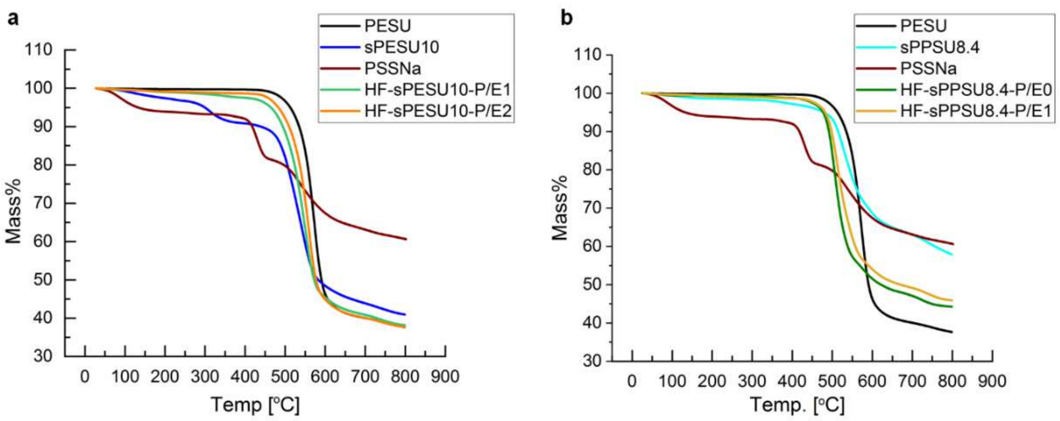

Figure S3. TGA of polymers, PSSNa and hollow fibers.

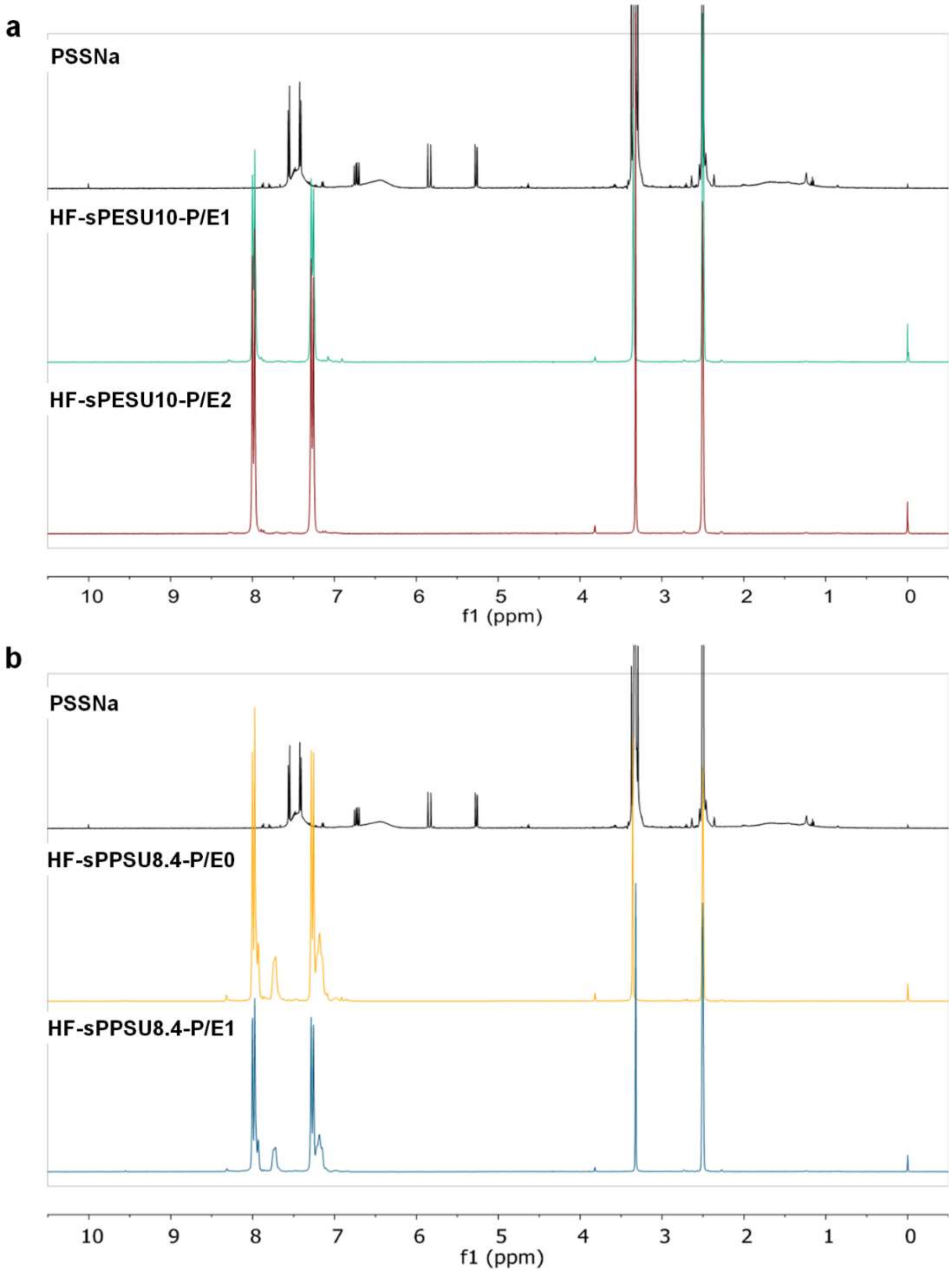

Figure S4. ^{1}H-NMR spectra of PSSNa and hollow fibers.

References

1. Guan, R.; Dai, H.; Li, C.; Liu, J.; Xu, J. Effect of casting solvent on the morphology and performance of sulfonated polyethersulfone membranes. *J. Memb. Sci.* **2006**, *277*, 148–156.
2. Li, Y.; Chung, T.S. Highly selective sulfonated polyethersulfone (spes)-based membranes with transition metal counterions for hydrogen recovery and natural gas separation. *J. Memb. Sci.* **2008**, *308*, 128–135.
3. Guan, R.; Zou, H.; Lu, D.; Gong, C.; Liu, Y. Polyethersulfone sulfonated by chlorosulfonic acid and its membrane characteristics. *Eur. Polym. J.* **2005**, *41*, 1554–1560.
4. Lufrano, F.; Gatto, I.; Staiti, P.; Antonucci, V.; Passalacqua, E. Sulfonated polysulfone ionomer membranes for fuel cells. *Solid State Ion.* **2001**, *145*, 47–51.

© 2018 by the authors. Submitted for possible open access publication under the terms and conditions of the Creative Commons Attribution (CC BY) license (http://creativecommons.org/licenses/by/4.0/).
